# A GCN2-Like eIF2α Kinase (LdeK1) of *Leishmania donovani* and Its Possible Role in Stress Response

**DOI:** 10.1371/journal.pone.0156032

**Published:** 2016-06-01

**Authors:** Shilpa J. Rao, Shimi Meleppattu, Jayanta K. Pal

**Affiliations:** 1 Cell and Molecular Biology Laboratory, Department of Biotechnology, Savitribai Phule Pune University, Ganeshkhind, Pune-411007, India; 2 Interdisciplinary School of Health Sciences, Savitribai Phule Pune University, Ganeshkhind, Pune-411007, India; Louisiana State University, UNITED STATES

## Abstract

Translation regulation in *Leishmania* parasites assumes significance particularly because they encounter myriad of stresses during their life cycle. The eukaryotic initiation factor 2α (eIF2α) kinases, the well-known regulators of translation initiation in higher eukaryotes have now been found to control various processes in these protozoan parasites as well. Here, we report on cloning and characterization of a GCN2-like eIF2α kinase from *L*. *donovani* and also on its modulation during nutrient starvation. We cloned a GCN2-like kinase from *L*. *donovani*, which we named as LdeK1 and validated it to be a functional eIF2α kinase by *in vitro* kinase assay. LdeK1 was found to be localized in the cytoplasm of the promastigotes with a five-fold higher expression in this stage of the parasite as compared to the axenic amastigotes. Phosphorylation of eIF2α and a G1-arrest was observed in response to nutrient starvation in the wild-type parasites. In contrast, phosphorylation was significantly impaired in a dominant-negative mutant of LdeK1 during this stress with a subsequent failure to bring about a G1-arrest during cell cycle. Thus, LdeK1 is a functional GCN2-like kinase of *L*. *donovani* which responds to nutrient starvation by phosphorylating its substrate, eIF2α and a G1-arrest in the cell cycle. Nutrient starvation is encountered by the parasites inside the vector which triggers metacyclogenesis. We therefore propose that global translational regulation by activation of LdeK1 followed by eIF2α phosphorylation and G1-arrest during nutrient starvation in the gut of sandfly vector could be one of the mechanisms to retool the cellular machinery required for metacyclogenesis of *Leishmania* promastigotes.

## Introduction

*Leishmania* spp. are protozoan parasites belonging to the family trypanosomatidae and are the causative agent of leishmaniasis. Among the various clinical forms of leishmaniasis that exist, the visceral leishmaniasis (VL) is the most debilitating to the public health. According to WHO reports, about 3 million people are estimated to be infected with VL and over 20,000 death occurs annually. *Leishmania donovani* is the species responsible for VL in India, Nepal and Bangladesh. These organisms have a dimorphic life-cycle wherein the flagellated, extracellular promastigote forms exist in the midgut of the sandfly vector and non-motile intracellular amastigote forms thrive in the macrophages of the mammalian host. Prior to infection of the mammalian host, the non-infective promastigote forms are transformed into the infective metacyclic forms (metacyclogeneis) [1,2]. The factors that trigger this process still remain to be identified. However, stress conditions like low pH, nutrient starvation, low levels of tetrahydrobiopterin and absence of purines were found to promote metacyclogenesis *in vitro*, though a clear picture of the *in vivo* condition is missing [3,4]. During blood meal, the infected sandfly releases the metacyclic forms of the promastigotes in the bloodstream where they encounter elevated temperature (37°C) followed by low pH in the phagolysosomal compartment of host macrophages. These environmental cues trigger differentiation of the parasites into the amastigotes (rev in [5]) that involves programmed retooling of gene expression [6] and drastic changes in proteomic profiles [7].

In absence of transcription control in *Leishmania*, gene regulation at the level of translation, gains importance. The eukaryotic initiation factor 2α (eIF2α) kinases are well-known regulators of translation in higher eukaryotes. eIF2 takes part in the initiation of protein synthesis in the form of a ternary complex (eIF2-GTP-Met-tRNAi) wherein the GTP gets hydrolyzed to GDP subsequently towards the end of initiation. eIF2α kinases undergo autophosphorylation, get activated in response to different stress conditions and subsequently phosphorylate the alpha-subunit of eIF2 at S-51 residue (rev in [8]). The phosphorylated form shows higher affinity towards eIF2B, a guanine nucleotide exchange factor which performs the GDP-GTP exchange during recycling of active eIF2B. Being in rate-limiting amount, the sequestered eIF2B is not available for the GDP-GTP exchange and thus eIF2 fails to carry out the next round of the initiation step, thereby inhibiting protein synthesis. Four eIF2α kinases have been described in mammals (rev in [9]). These are, the heme-regulated inhibitor (HRI) which gets activated under heme deficiency and several other conditions, the double-stranded RNA activated protein kinase (PKR) which gets activated during viral infection, the PKR-like endoplasmic reticulum-resident kinase (PEKR) which gets activated during the presence and accumulation of unfolded proteins in the endoplasmic reticulum and the general control non-derepressible 2 (GCN2) kinase which gets activated during nutrient starvation [10,11].

Translation regulation by eIF2α kinases have also been reported in protozoan parasites. In *Plasmodium falciparum*, PfeIK1 was reported to regulate stress response to amino acid starvation [12]. The other kinase IK2 was shown to control the latency of sporozoites in the mosquito salivary gland [13]. The third eIF2α kinase, PfPk4 was found to be associated with the arrest of global protein synthesis in schizonts and gametocytes enabling completion of the erythrocytic cycle of *P*. *falciparum* [14,15]. In *Toxoplasma gondii*, the kinase TgIF2K-A was reported to be the regulator of unfolded protein response located in endoplasmic reticulum and another kinase TgIF2K-B was shown to be possibly involved in cytoplasmic stress [16]. In *T*. *gondii*, phosphorylation level of eIF2α was demonstrated to be enhanced during *in vitro* differentiation from trachyzoites to bradyzoites indicating the role of eIF2α kinases in differentiation process [17] and in maintaining the quiescent state of bradyzoites [16]. A GCN2-like eIF2α kinase, TgIF2K-D was reported to be involved in the extracellular survival of the *T*. *gondii*. However, it was found to be unnecessary during nutrient starvation [18,19]. Three eIF2α kinases have been reported in *Tryanosoma bruce*i of which TbIF2K2 was shown to be located in the flagellar pocket, however its function is yet to be determined [20]. In *Trypanosoma cruzi*, eIF2α phosphorylation was found to be a prerequisite for metacyclogenesis [21]. In *L*. *infantum*, it is suggested that phosphorylation of eIF2α is an important event during differentiation which gets delayed in the absence of a PERK-like kinase [22]. Further studies using polysome profiling suggest the involvement of translation regulation by eIF2α phosphoylation during promastigote to amastigote differentiation. The phosphorylation of eIF2α causes downregulation in global protein synthesis, however, it supports the upregulation of developmentally regulated genes in *L*. *infantum* necessary for differentiation of the parasites [23].

In the present study, we have cloned and characterized an eIF2α kinase from *L*. *donovani* that shows similarity to GCN2 kinase of other eukaryotes. We named this kinase as LdeK1. Further, by generation of a dominant-negative LdeK1 mutant of *L*. *donovani*, we demonstrate that LdeK1 is the eIF2α kinase that is involved during nutritional stress. Unlike the wild-type parasites, the mutants failed to phosphorylate eIF2α and bring about a G1-arrest in the cell cycle. We hypothesize that nutrient starvation, which triggers metacyclogenesis in promastigotes, activates LdeK1 which phosphorylates eIF2α followed by a cell-cycle arrest in the G1-phase. All these events might lead to remodeling of the cellular dynamics in the parasite, facilitating their transformation process.

## Materials and Methods

### Ethics statement

This study was carried out in strict accordance with the recommendations in the Guide for the Care and Use of Laboratory Animals of the National Institute of Health. The protocol was approved by the Institutional Animal Ethics Committee of the Savitribai Phule Pune University (Permit number; 538CPCSEA). All surgeries were performed under sodium pentobarbital anesthesia, and all efforts were made to minimize suffering.

### *Leishmania* culture

Promastigotes of *L*. *donovani* strain (MHOM/IN/1983/AG83) were obtained from the cell repository, National Centre for Cell Science, Pune, India and cultured in M199 (Sigma, St. Louis) supplemented with 10% Foetal Bovine Serum (FBS) (Sigma, St. Louis) at 25°C. The culture was maintained by sub passaging the parasites every fifth day *in vitro*. Axenic amastigotes of *L*. *donovani* were generated as described by Saar *et al*. [24]. Briefly, late-log phase promastigotes were cultured in M199 supplemented with 20% FBS at 37°C and 5% CO_2_ for 24 h. Later, the culture was maintained in the medium at a low pH (pH 5.5) wherein the differentiation is completed in 120 h.

### Cloning, overexpression and purification of recombinant proteins

Genomic DNA was isolated from *L*. *donovani* promastigotes using genomic DNA isolation kit (Chromous Biotech, India) as per the manufacturer’s protocol. The genomic DNA was used as the template for all subsequent PCR amplifications using phusion DNA polymerase (Finzymes, Thermo). The primer sequences and restriction sites used are mentioned in Table 1. The catalytic domain of LdeK1 named as LdeK1KD (1164 bp), its substrate, LdeIF2α (1299 bp) and N-terminal segment of LdeK1 named as LdeK1NT (546 bp) were PCR amplified with primer pairs 2, 3, and 4, respectively and were cloned in pET28a vector (Novagen) with appropriate restriction enzymes using T_4_ DNA ligase (Fermentas). Transformations of the ligated products were carried out in *E*.*coli* (JM109 strain) and the positive colonies were screened by the release of the insert from the isolated plasmids by restriction digestion analysis. The clones were further confirmed by DNA sequencing. To generate a mutant form of LdeIF2α (LdeIF2αT166A), the clone of pET28a-LdeIF2α was mutated by site-directed mutagenesis (Quick change II Site-Directed Mutagenesis Kit, Agilent Technologies) using primer pair 5 mentioned in Table 1. For overexpression of recombinant proteins (LdeK1KD, LdeIF2α and LdeK1NT), the cloned plasmids were transformed in *E*. *coli* BL21 Rosetta cells and expression was induced using 1mM IPTG (Sigma). The purification and refolding were carried out as described previously [25].

**Table 1:** List of primers.

Primers (5′-3′)
Fp1 GCTGGAATTGATGGCCAAAAAG
Rp1 TCCTGCTTATTTTTCTTGTGGG
Fp2 ACCGG*GAATTC*TTCATTCAGCAGCG
Rp2 GAAGC*GCGGCCGC*CGCTCAGCACAG
Fp3 CGAA*CATATG*GCTTCTTACTGTGTCA
Rp3 GTGACT*GTCGAC*GTCCGCATCCTCATC
Fp4 TACGAGGTA*GAATTC*ATGGCCAAGGAG
Rp4 ACGCTG*CTCGAG*GAACTCGTTCC
Fp5 CCGTACACGGAGGTGGCTAGACGCCGCGTGTCG
Rp5 CGACACGCGGCGTCTAGCCACCTCCGTGTACGG
Fp6 AAGGCGAAGCAGATCAGGTG
Rp6 GAAAGGCTGCGGACAGTTTG
Fp7 CCTACCATGCCGTGTCCTTCTA
Rp7 AACGACCCCTGCAGCAATAC
Fp8 TGCTGA*GATCT*ATGGCCAAAAAG
Rp8 GTTCA*GATCT*CGACCCTCTGAACC
Fp9 TCTGCGTTTCACTTGCTCTC
Rp9 TGCTGGCATTTTGTCCAATTG

### *In vitro* eIF2α kinase assay

*In vitro* eIF2α kinase assay was performed as described by Chen *et al*. [26] with some modifications. Briefly, the purified and *in vitro* refolded 2.5 μg of LdeK1KD was incubated with either 1 μg of LdeIF2α or 1μg of human eIF2α (hEIF2α) along with kinase assay buffer (20 mM Tris–HCl, pH 7.6, 2 mM magnesium acetate, 40 mM KCl) and 0.5 mM ATP at 30°C for 30 min. Lysates of Rosetta cells both untransformed and transformed with pET28a vector were used as controls to exclude the possibility of a contaminating kinase in the preparation. The reaction was stopped by addition of 5X Laemmli sample buffer and was subjected to 10% SDS PAGE followed by electrotransfer to nitrocellulose membrane [27]. The blots were probed with anti-phospho-eIF2α antibody (Invitrogen) and HRP-conjugated anti-rabbit secondary antibody (Sigma) following which they were stripped and reprobed with anti-total eIF2α antibody. The bands were visualized using chemiluminescence detection kit (Roche).

#### Real Time PCR

Total RNA was extracted from *L*. *donovani* promastigotes and axenic amastigotes using TRI^®^ reagent (Sigma) and was quantified spectrophotometrically. 2 μg of RNA was used for first-strand cDNA synthesis (High capacity cDNA Reverse Transcription kit, ABI). Quantitative Real Time PCR was performed using Stepone Real Time PCR system in triplicates. Briefly, a 10μl reaction contained 1X power SYBR^®^ green PCR master mix (ABI), 200 nM of each forward and reverse primers 6 and 7 (Table 1) and 2.5 μl of cDNA. The PCR amplification cycle consisted of initial denaturation at 95°C for 10 min, followed by 40 cycles of 95°C for 15 sec, 55°C for 20 sec and 72°C for 20 sec. The relative expression level of LdeK1 transcripts was normalized to that of *rRNA45* gene [28] used as the endogenous control by comparative Ct method [29].

### Nutrient starvation

Wild-type and the dominant-negative mutant of *L*. *donovani* promastigotes were washed with Earle’s balanced salt solution (EBSS, Sigma) and starved in EBSS for 1 h, 2 h, 4 h, 6 h and 24 h. Parasites starved for 4 h were recovered in complete media for 24 h. Parasites were then harvested and used for western blotting and cell cycle analysis.

### Generation of *L*. *donovani* dominant-negative mutant of LdeK1

To generate a *L*. *donovani* dominant-negative mutant (DNM), the DNA fragment representing the N-terminal segment (1-840bp) of LdeK1 was PCR amplified with primer pair 8 carrying BglII restriction sites. The fragment was cloned in pIR1SATvector at the BglII site followed by screening of positive clones. The cloned plasmid was digested with SwaI and the linear fragment was gel purified. 10 ug of DNA was used for electroporation by high voltage protocol as described by Robinson *et al*. [30]. Nourseothricin (Sigma) (50μg/ml) was added to the culture, 24 h after electroporation and cloned by limiting dilutions. Single parasite clones were screened by PCR for integration of the DNA fragment representing the N-terminal segment of LdeK1 in the *Leishmania* genome using primer pair 9 as mentioned in Table 1. The overexpression of the N-terminal segment of LdeK1 in *L*. *donovani* at the transcript level was confirmed by RT-PCR using primer pair 8 as mentioned in Table 1 and at protein level by western blot using anti-LdeK1 antibody.

### Polyclonal antibody production

The recombinant, purified and refolded LdeIF2α protein was used for polyclonal antibody production in New-Zealand rabbit. 7 days prior to immunization, blood was withdrawn from rabbit’s central ear vein and processed for collection of the pre-immune serum. 100 μg of protein along with Freund’s complete adjuvant was injected subcutaneously at four sites followed by booster doses in Freund’s incomplete adjuvant at day 14 and day 30. On day 37, blood was collected and processed for serum isolation. The purified recombinant N-terminal segment of LdeK1 was used for custom-made polyclonal antibody production (Bangalore genei, India).

### Western blotting

Promastigotes and amastigotes of *L*. *donovani* were harvested, washed in PBS and lysed by sonication using protein extraction buffer containing 20 mM Tris-Cl, 1 mM EDTA, 0.1% Triton X-100, 1mM phenymethylsuphonylfluoride, protease inhibitor cocktail (Roche) and Phosstop (Roche). The supernatant containing soluble protein was obtained by centrifugation at 14000 g for 40 min at 4°C. The proteins were quantified by Bradford’s assay and equal quantities of proteins were separated by 8–10% SDS PAGE. The proteins were electrotransferred onto nitrocellulose membranes. For phospho-eIF2α and total eIF2α, blocking was performed in 5% BSA in TBST for 1 h at RT whereas for LdeK1 it was performed in 2% BSA in TBST for overnight at 4°C. The blots were probed with anti-phospho-eIF2α antibody (Invitrogen) at 1:1000 dilution for overnight at 4°C, for anti-total-eIF2α antibody and anti-LdeK1 antibody at 1:1000 dilution for 1h at RT. The blots were washed and incubated with HRP-conjugated anti-rabbit secondary antibody (Sigma) at a dilution of 1:2500 for 1 h at RT. The bands were visualized using chemiluminescence detection kit (Roche) and densitometric analysis carried out using ImageJ software.

### Immunofluorescence studies

Log-phase promastigotes were fixed in 4% paraformaldehyde for 15 min followed by washing (in PBS) and permeabilization in 0.1% Triton X-100 (in PBS) for 15 min. The parasites were allowed to adhere on to poly-L-lysine coated slides for 1 h. The parasites were then blocked in 2% BSA (in PBS) followed by incubation with anti-LdeK1 antibody (1:200) for 1 h and cy3-conjugated anti-rabbit secondary antibody (Millipore) for 1 h. DABCO (Sigma) was used as an anti-fading agent. Images were acquired using a confocal laser scanning microscope (Ziess LSM510 Meta, Belgium).

### Cellular Fractionation and localization of LdeK1

Promastigotes were subjected to subcellular fractionation by digitonin (Sigma) as described previously by Chow *et al*. [22]. The proteins obtained in the soluble (cytosolic, F1) and the insoluble fraction (F2) including the organelle fraction was analyzed by western blotting using anti-tubulin antibody (Sigma) and anti-LdeK1 antibody.

### Cell cycle analysis

10^7^ promastigotes of *L*. *donovani*, both wild-type and the dominant-negative mutant were subjected to nutrient starvation as described above. These parasites were washed with ice-cold PBS twice followed by fixation in ice-cold 70% methanol for overnight at -20°C. The parasites were then centrifuged at 1300g for 10 min followed by washing and resuspension in ice-cold PBS. The parasites were subjected to DNAase free RNAse treatment (50 μg/ml) at 37°C for 30 min followed by staining with propidium iodide (50 μg/ml). The stained cells were analysed by FACSCalibur fluorescence activated cell sorter using CellQuest Pro software (Becton Dickinson, USA). 10,000 events per sample were analyzed for determination of cell cycle distribution.

### Detection of nascent (*de novo*) protein synthesis

Wild-type and the dominant-negative mutant promastigotes were subjected to nutrient starvation in EBSS for 6 h and 24 h to analyze its effect on nascent protein synthesis by non-radioactive method involving Click chemistry (Invitrogen). The parasites (both control and stressed) were incubated in methionine-free media for 30 min. It was later replaced with methionine-free media containing 50 μM of the methionine-analog, L-azidohomoalanine (AHA) for 3 h allowing incorporation of AHA into nascent proteins. After incubation the cells were lysed using 50 mM Tris, pH 8.0 containing protease inhibitor and PMSF with a sonicator. The lysate containing the AHA-incorporated nascent proteins were crosslinked to TAMRA alkyne (Invitrogen) as per the manufacturer’s protocol. The labelled protein were loaded on a SDS-Polyacrylamide gel followed by imaging in Typhoon 9400 laser scanner (G.E., Healthcare).

### Giemsa staining for morphological analysis

Log-phase promastigotes both wild type and mutant were subjected to nutrient starvation as described earlier. The parasites were fixed in methanol followed by Giemsa staining for morphological analysis.

## Results

### LdeK1 is a putative GCN2-like eIF2α kinase of *L*. *donovani*

BLAST search using the catalytic domain of eIF2α kinases from mammals revealed the presence of three ORFs of eIF2α kinases in *L*. *donovani* database [31]. One of the kinases LdBPK_11.0060 is a potential homolog of GCN2 of higher eukaryotes. We have cloned this GCN2-like kinase from *L*. *donovani* (AG83 strain) and named it as LdeK1 (S1 Fig) with a GeneBank accession number, KP325123. The open reading frame (ORF) of LdeK1 is of 3687 bp encoding a putative 1228 amino acid product. It is in contrast to a shorter ORF annotated in *L*. *donovani* database, as *in silico* analysis predicts that a RWD domain, characteristic of this kinase, is present upstream of the annotated start site. The 5′- and 3′-ends of the 3687 bp ORF were further confirmed by 5′- and 3′- RACE analysis which showed a 5′-UTR of 82 bp and a 3′- UTR of 794 bp, respectively.

**Fig 1:**
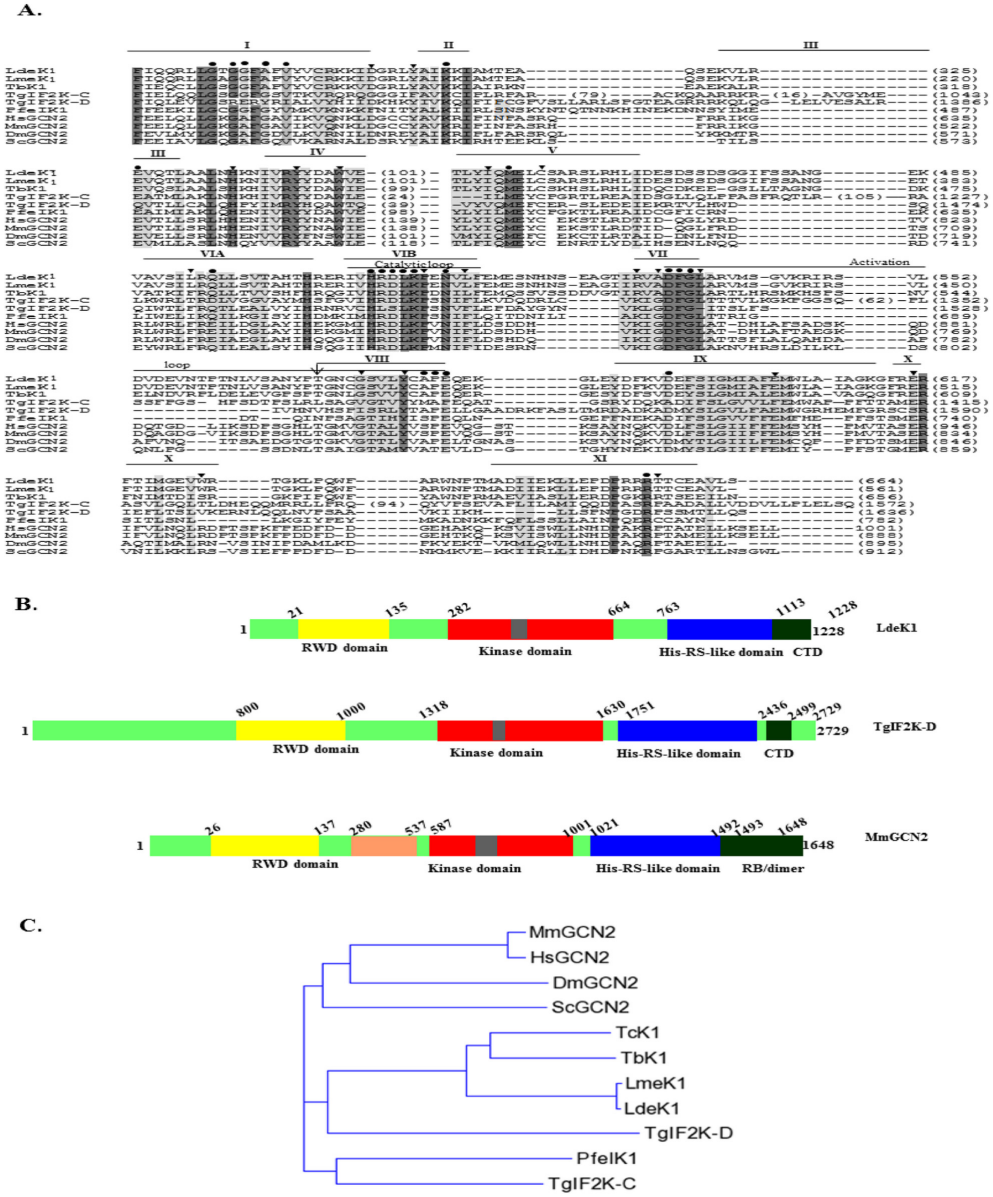
An eIF2α kinase (LdeK1) homologous to GCN2 is present in *L*. *donovani*. (**A**) CLUSTAL W analysis of the catalytic domain of LdeK1 from *L*. *donovani* (KP325123) with GCN2 of *Homo sapiens*, HsGCN2 (NP_001013735); *Mus musculus*, MmGCN2 (NP_001171277); *Saccharomyces cerevisia*e, ScGCN2 (AAA34636); *Drosophila melanogaster*, DmGCN2 (AGB96521); *Leishmania major*, LmeK1 (XP_001681474); *Trypanosoma brucei*, TbK1, (XP_828792); *Plasmodium falciparum*, PfeIK1 (XP_001348597), *Toxoplasma gondii*, TgIF2K-C (AHM92904); TgIF2K-D (AED01979). The catalytic domain comprises of I-IX subdomains which are demarcated by solid black lines on top. Number between subdomain IV and V represents the variable length of the kinase insert present in different kinases. The kinase insert sequence has been omitted to facilitate the alignment. Identical residues are indicated in dark grey and similar sequences are highlighted in light grey. Dots represent residues conserved in kinases and shaded triangles indicate residues that are specific to eIF2α kinases. The arrow in subdomain VIII indicates the residue that is autophosphorylated during activation of PKR. (**B**) Domain organization of LdeK1 along with TgIF2K-D and MmGCN2 for comparison. The numbers demarcate the residue numbers for each domain of the proteins. **(C)** Phylogenetic analysis of LdeK1.

Multiple alignment of the catalytic domain of LdeK1 (282–664 residues) with the catalytic domain of other known eIF2α kinases (Fig 1A) revealed the presence of all the eleven subdomains with a characteristic kinase insert sequence (101 residues) between subdomains IV and V, which is known to be of variable length in other eIF2α kinases. Further, a highly conserved motif in subdomain IV (LYIQMELC) identified in all eIF2α kinases and a lysine residue equivalent to lysine 296 of PKR is also found in LdeK1. The conserved threonine residues which are involved in the autophosphorylation and activation of eIF2α kinases are found to be conserved in this kinase also. Pfam motif search revealed the presence of a RWD domain (21–135 residues) in LdeK1 (Fig 1B) with an E-value of 7.4e-07, a feature highly conserved among most of the GCN2 orthologs [32,33]. The RWD domain interacts with GCN1-GCN20 complex, required for optimum activation of the kinase [34]. The *L*. *donovani* genome also encodes a putative GCN1 (XP_003860095.1) and a less conserved putative GCN20 indicating the possibility of existence of LdeK1-GCN1-GCN20 association in the parasites. Other homologous proteins like, YIH and IMPACT in yeast and humans, respectively, interact with the same fragment of GCN1 that binds to GCN2 and therefore inhibit the kinase. A homolog of this protein (XP_003865631.1) is also found in *L*. *donovani* genome as predicted by *in silico* analysis with a significance value of 3e-14. However, LdeK1 lacks the pseudokinase domain whose occurrence in other GCN2 orthologs is known to contribute to the activation of the kinase by uncharged tRNA [35–37]. GCN2-like kinases of other protozoan parasites including TgIF2K-C/D of *T*. *gondii* and PfeIK1 of *P*. *falciparum* also lack this domain [12,19,38]. Another evolutionary conserved feature of GCN2 is the presence of the histidyl-tRNA sythetase (HisRS) domain that recognizes the uncharged tRNA in amino acid starved cells [10,39,40]. HHpred analysis predicts a HisRS domain (763–1113 residues) in LdeK1 (Fig 1B) with a histidine B sequence (AFGCGLD), characteristic of HisRS-related domain [38, 40,41] despite very low conservation at the sequence level. This weak conservation is a common feature observed in other protozoan parasites [12,19] probably due to their early divergence in the course of evolution. The C-terminal domain (CTD) of LdeK1 harbors clusters of basic and hydrophobic amino acids which in yeast GCN2 were found to be required for its association with rRNA of ribosomes and thereby regulate the translation machinery [42,43]. Phylogenetic analysis suggests that LdeK1 is related to the GCN2 homologs identified from other organisms and evolutionarily more close to GCN2 like kinases identified from protozoan parasites. *T*. *brucei* and *T*. *cruzi* have been added as outgroup in the analysis. (Fig 1C).

### LdeK1 is a functional eIF2α kinase of *L*. *donovani*

**Fig 2:**
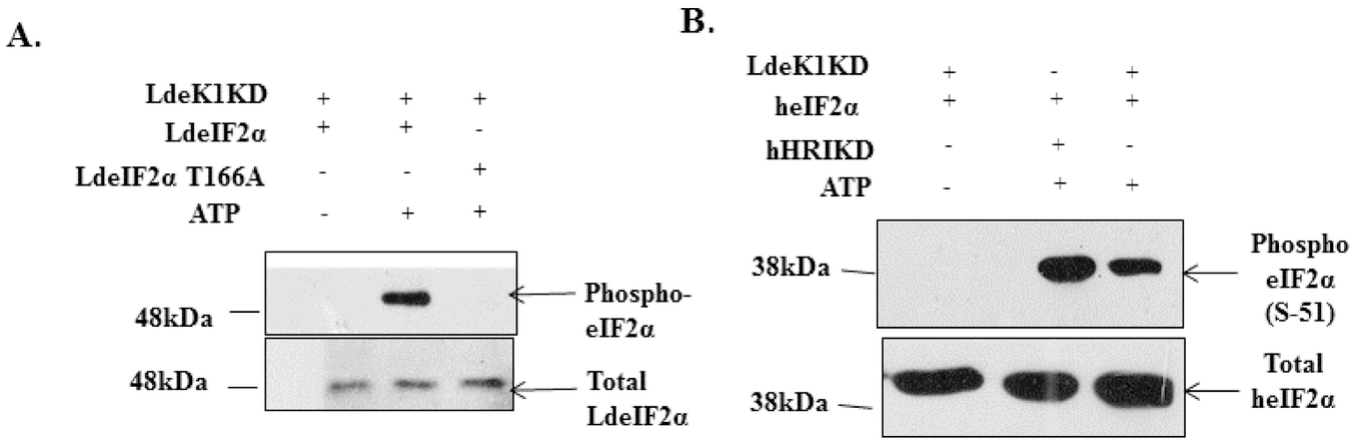
LdeK1 is a functional eIF2α kinase of *L*. *donovani* that phosphorylates LdeIF2α at T-166 residue *in vitro*. The bacterially overexpressed catalytic domain of LdeK1 (LdeK1KD) was incubated with **(A)** wild-type LdeIF2α and LdeIF2α T166A **or** heIF2α (**B**) along with cold ATP at 30°C and subjected to SDS-PAGE. As a positive control, the bacterially overexpressed catalytic domain of human heme-regulated inhibitor (hHRIKD) was incubated with heIF2α along with cold ATP at 30°C and subjected to SDS-PAGE. The phosphorylation of eIF2α was analyzed by western blotting using anti-phospho-eIF2α antibody. (**A**) Leishmanial total eIF2α (LdeIF2α) and (**B**) human total eIF2α (heIF2α) were used as loading controls using *Leishmania*-specific and human-specific eIF2α antibodies, respectively. LdeK1 could phosphorylate LdeIF2α and not LdeF2αT166A indicating LdeK1 to be a functional eIF2α kinase capable of phosphorylating LdeIF2α at threonine-166 residue and heIF2α at serine-51 residue.

To validate the putative ORF of LdeK1 as a functional eIF2α kinase, an *in vitro* eIF2α kinase assay was performed. The catalytic domain of eIF2α kinases was found to be sufficient for their eIF2α kinase activity *in vitro* [22,25]. For this purpose, the catalytic domain of LdeK1 (LdeK1KD) and its substrate, LdeIF2α were cloned, overexpressed in *E*.*coli*, purified and *in vitro* refolded (S2 Fig). The LdeK1KD was incubated with LdeIF2α in presence of ATP and the phosphorylation of the substrate was analyzed by western blotting using anti-phopsho-eIF2α and anti-total eIF2α antibodies. The LdeK1KD was capable of phosphorylating the substrate, however was unable to phosphorylate the mutant substrate LdeIF2αT166A, where threonine-166 was replaced by alanine, confirming that threonine-166 is the site of phosphorylation of LdeIF2α by eIF2α kinases in *L*. *donovani* (Fig 2A). Interestingly, the LdeK1KD was also able to phosphorylate the human eIF2α (heIF2α) at S-51 residue as analyzed by western blotting using human phospho-eIF2α antibody (Fig 2B). Further, the bacterially overexpressed kinase domain of human heme-regulated inhibitor (hHRIKD) phosphorylated heIF2α which was used as a positive control. These results indicate that the LdeK1 is a functional eIF2α kinase, belonging to serine-threonine family of kinases. To exclude the possibility of any contaminating kinase in the bacterially overexpressed LdeK1KD preparations, an *in vitro* kinase assay with *E*.*coli* (Rosetta) lysates that were untransformed and transformed with pET28a vector were performed. No signal was however detected by western blotting when either of the lysates was used as a source of possible contaminating kinase (S3 Fig).

### LdeK1 is expressed in the promastigote form of *L*. *donovani*

**Fig 3:**
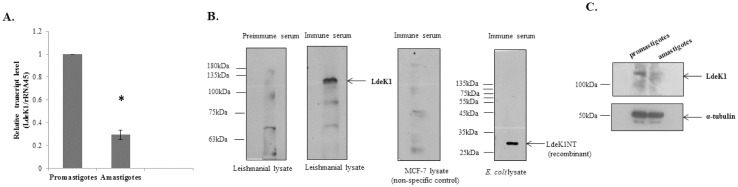
Expression of LdeK1 in *L*. *donovani*. **(A)** LdeK1 is mainly expressed in the promastigotes of *L*. *donovani* at the mRNA level. Transcript levels of LdeK1 was analysed by quantitative real-time PCR in both *L*. *donovani* promastigotes and axenic amstigotes. The transcript level was approximately 5 fold more in promastigotes as compared to the axenic amastigotes.*p < 0.005 **(B)** Immunoblot to analyze the specificity of the antibody raised against LdeK1. The N-terminal domain of LdeK1 was bacterially overexpressed and partially purified followed by antibody production. The antibody could detect the source protein purified from *E*. *coli* and a protein at approximately 130 kDa in total leishmania lysates using western blotting. No protein was detected in *Leishmania* lysates with pre-immune sera and in MCF-7 lysates with immune sera, used to test the species specificity of the antibody. **(C)** Western blot analysis using anti-LdeK1 antibody revealed the expression of LdeK1 in the promastigote stage of the parasite. α-tubulin was used as a loading control.

To analyze the expression of LdeK1 in two different life-stages of *L*. *donovani*, a Real-Time PCR using LdeK1-specific primers was performed. The data showed a 5-fold increase in the transcript levels of LdeK1 in promastigotes as compared to the axenic amastigotes (Fig 3A). A polyclonal antibody was developed against an N-terminal segment LdeK1 (LdeK1NT) whose specificity was confirmed by western blot analysis as it could recognize the bacterially overexpressed LdeK1NT protein (Fig 3B). Further, the antibody detected a band of approximately 130 kDa in the lysates of *Leishmania* promastigotes, the lysates of MCF-7 was used as the negative control (Fig 3B). Western blot analysis using the above antibody indicated expression of LdeK1 protein in the promastigotes. (Fig 3C).

### LdeK1 is localized in the cytoplasm of *L*. *donovani*

**Fig 4:**
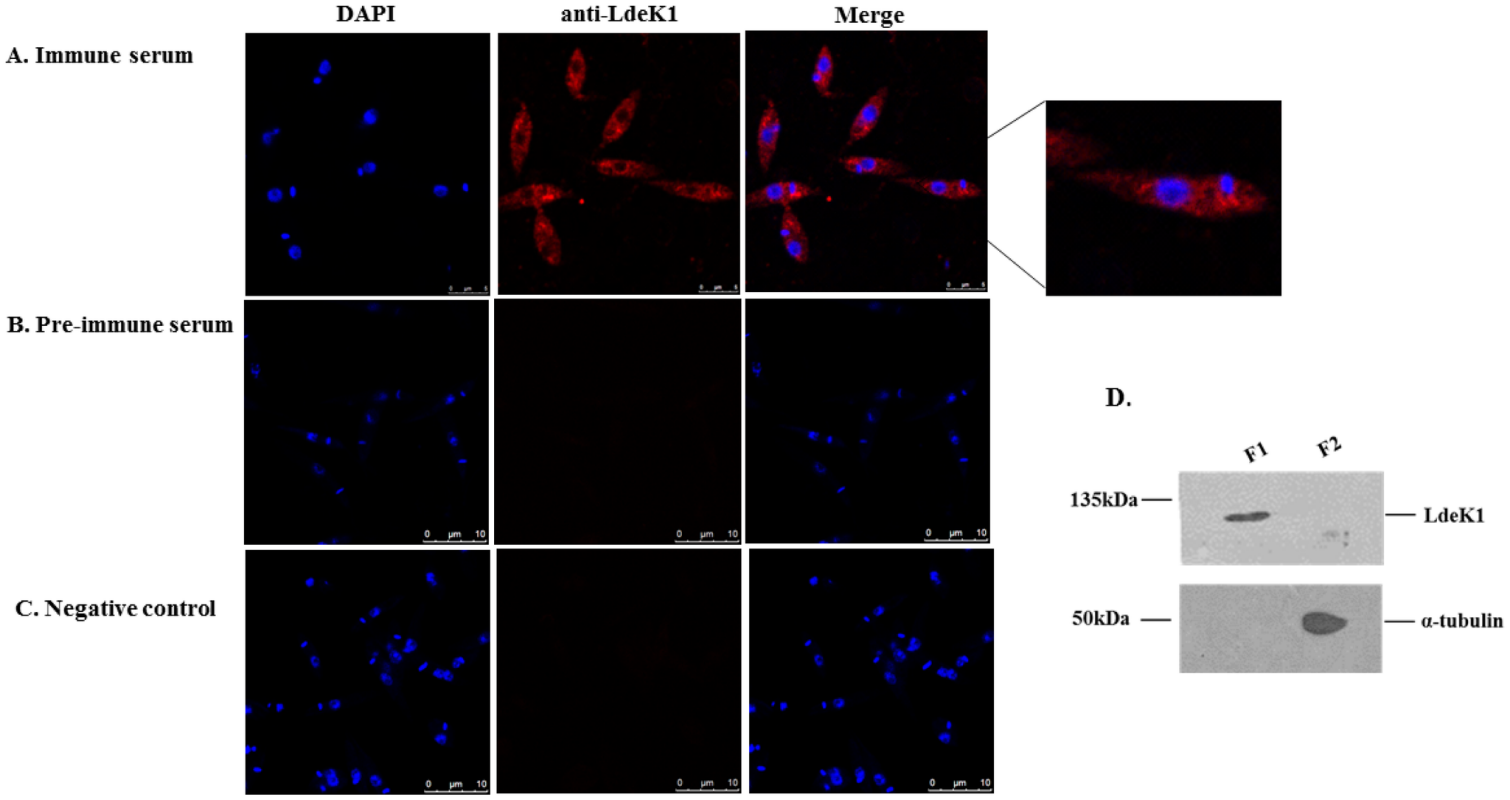
LdeK1 is localized in the cytoplasm of *L*. *donovani* promastigotes. **(A.)** Indirect immunofluorescence was performed using anti-LdeK1 antibody (immune-sera) and cy3-conjugated secondary antibody (red) to detect the localization of the LdeK1 in promastigotes of *L*. *donovani*. Nuclear DNA was stained with DAPI (blue). LdeK1 was found to localize in the cytoplasm of promastigotes. **(B.)** preimmune serum did not give any signal. **(C.)** negative control, stained with cy3-conjugated secondary antibody alone. **(D)** Cellular fractionation of promastigotes using digitonin resulted in a soluble cytosolic fraction (F1) and insoluble fraction including the organelle fraction (F2). The LdeK1 was detected in the F1 fraction by anti-LdeK1 antibody. α-tubulin was used as control for the cytoskeleton bound insoluble fraction.

Indirect immunofluorescence by confocal microscopy using anti-LdeK1 antibody revealed that LdeK1 localizes in the cytoplasm of *L*. *donovani* promastigotes (Fig 4A). No signal was however obtained on staining the parasites with anti-rabbit secondary antibody alone (Fig 4B) or with pre-immune sera indicating the specificity of the fluorescence by anti-LdeK1 antibody (Fig 4C). Further, cellular fractionation of *Leishmania* using digitonin confirms the presence of LdeK1 in the soluble cytosolic fraction (F1) by western blot analysis using anti-LdeK1 antibody. The soluble cytosolic fraction was differentiated from the insoluble fraction (F2) by the absence of α-tubulin in F1 fraction and presence of it in the F2 fraction (Fig 4D).

### Dominant-negative *L*. *donovani* mutant for LdeK1

**Fig 5:**
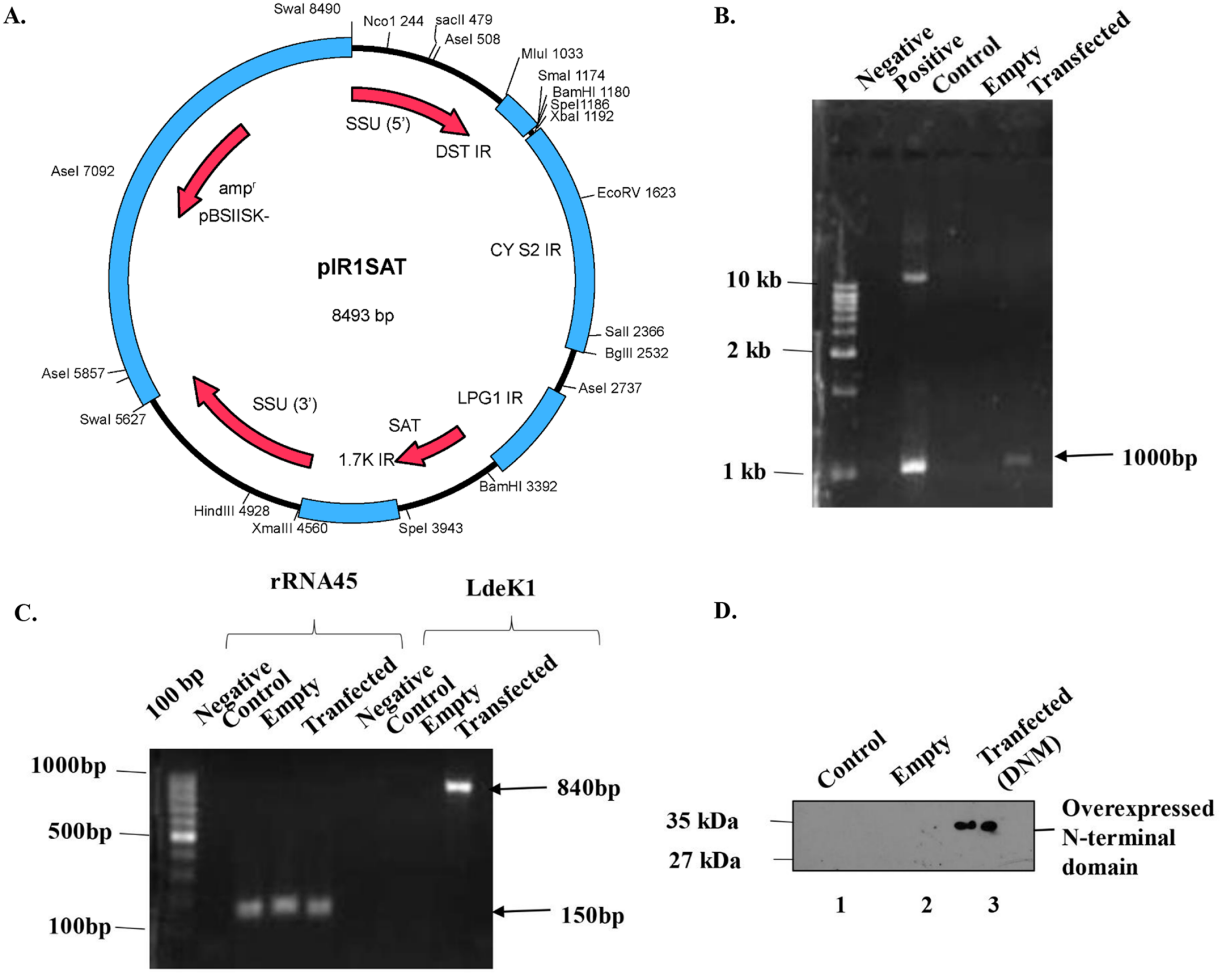
Generation of dominant-negative mutant of *L*. *donovani*. **(A)** Schematic representation of pIR1SAT vector used for generation of dominant negative mutant. The BglII was used for cloning of N- terminal domain of LdeK1. **(B)**. Integration of the DNA fragment representing the N-terminal domain of LdeK1 in the genome of *L*. *donovani*. PCR using genomic DNA from untransfected and transfected (empty and test) *L*. *donovani* detected a 1000 bp band indicating the integration of the required fragment in the genome of *L*. *donovani*. **(C)** Overexpression of N-terminal segment of LdeK1 in *L*. *donovani* at mRNA-level. The overexpression of N-terminal segment of LdeK1 at the transcript level was analysed by RT-PCR which shows the overexpression in transfected *Leishmania* but not in *Leishmania* transfected with empty vector or untransfected *Leishmania*. **(D)** Overexpression of N-terminal domain of LdeK1 in *L*. *donovani* at protein level. The overexpression of N-terminal domain of LdeK1 at the protein level was analyzed by western blotting using anti-LdeK1 antibody which shows the overexpressed protein in transfected *Leishmania* [dominant negative mutant (DNM); lane 3] but not in *Leishmania* transfected with empty vector (lane2) or untransfected *Leishmania* (control; lane 1).

In yeast, it has been reported that overexpression of the N-terminal segment of GCN2 generated a dominant-negative phenotype resulting in the loss of activation of endogenous GCN2 [34]. The overexpressed N-terminal segment of GCN2 competes with GCN1, a co-activator of GCN2, thereby keeping the kinase in an inactivated state. A similar strategy was adopted to generate a dominant-negative mutant of *L*. *donovani* for LdeK1 wherein the N-terminal segment of LdeK1 (1–281 residues) was overexpressed in *L*. *donovani* using pIR1SAT vector (Fig 5A). The integration of DNA fragment representing the N-terminal segment of LdeK1 in the genome of *L*. *donovani* was analyzed by PCR which showed integration in the parasites transfected with cloned vector, however genomic DNA of parasites transfected with the empty vector did not give any PCR amplification (Fig 5B). The overexpression of the N-terminal segment of LdeK1 in *L*. *donovani* was analyzed at the RNA level by RT-PCR which showed its overexpression in the parasites transfected with cloned vector. However, parasites transfected with the empty vector or the control parasites did not show any expression (Fig 5C). The overexpression of N-terminal segment was further confirmed at the protein level by western blotting using anti-LdeK1NT antibody which detected a protein of 35 kDa in the parasites transfected with cloned vector, however it did not show any expression in parasites transfected with empty vector or the control parasites (Fig 5D). The mutant parasites were viable with no significant change in the morphology and growth as compared to the wild type parasites (data not shown).

### eIF2α is phosphorylated during nutrient starvation in *L*. *donovani* and LdeK1 is the kinase involved in this response

**Fig 6:**
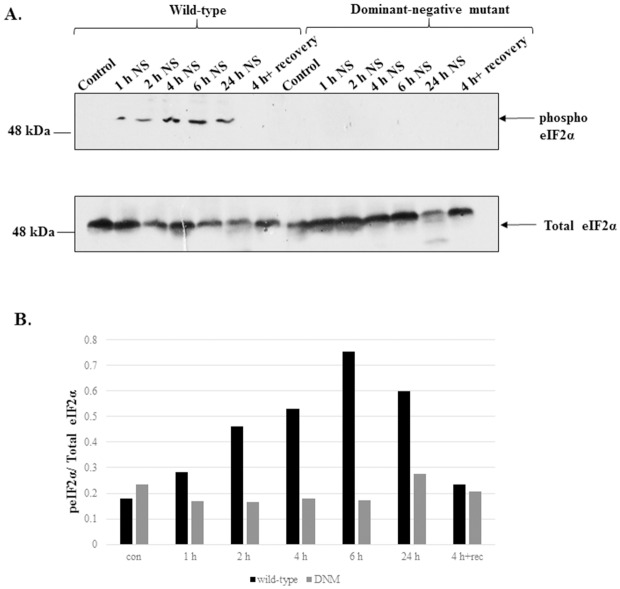
LdeK1 is involved during nutrient starvation to *L*. *donovani*. **(A)** Both wild type and the dominant-negative mutant (DNM) parasites were subjected to nutrient starvation for 1 h, 2 h, 4 h, 6 h and 24 h in EBSS. Parasites starved for 4 h were also recovered in complete media for 24 h. Total protein lysates were subjected to SDS-PAGE and electrotransferred. The phosphorylation was analyzed by western blotting using phospho-eIF2α antibody. Phosphorylation of eIF2α occurs in response to nutrient starvation in wild-type *L*. *donovani* parasites. However it is not seen in case of dominant-negative mutants (DNM) indicating the role of LdeK1 in nutrient starvation condition. Total eIF2α was used as a loading control. **(B)** Densitometric analysis of the western blot profile (peIF2α/total eIF2α).

*L*. *donovani* promastigotes were subjected to nutrient starvation for different time intervals namely, 1 h, 2 h, 4 h, 6 h and 24 h. The phosphorylation of eIF2α was analyzed by western blotting using anti-phospho-eIF2α antibody. As seen in Fig 6, the level of eIF2α phosphorylation increases with increasing time duration of nutrient starvation. However when parasites were recovered in the complete medium after 4 h of nutrient starvation, dephosphorylation of eIF2α was seen indicating the role of phosphatases during recovery from stress. To determine the specific eIF2α kinase that is involved in this response, the dominant-negative mutant parasites of *L*. *donovani* for LdeK1 were subjected to nutrient starvation. As seen in Fig 6A, the mutants were unable to phosphorylate eIF2α in response to nutrient starvation which suggests that LdeK1 is the kinase involved in this stress. The quantitative profile of eIF2α phosphorylation is shown in Fig 6B.

### G1-arrest occurs in response to nutrient starvation to *L*. *donovani* which is dependent on LdeK1

**Fig 7:**
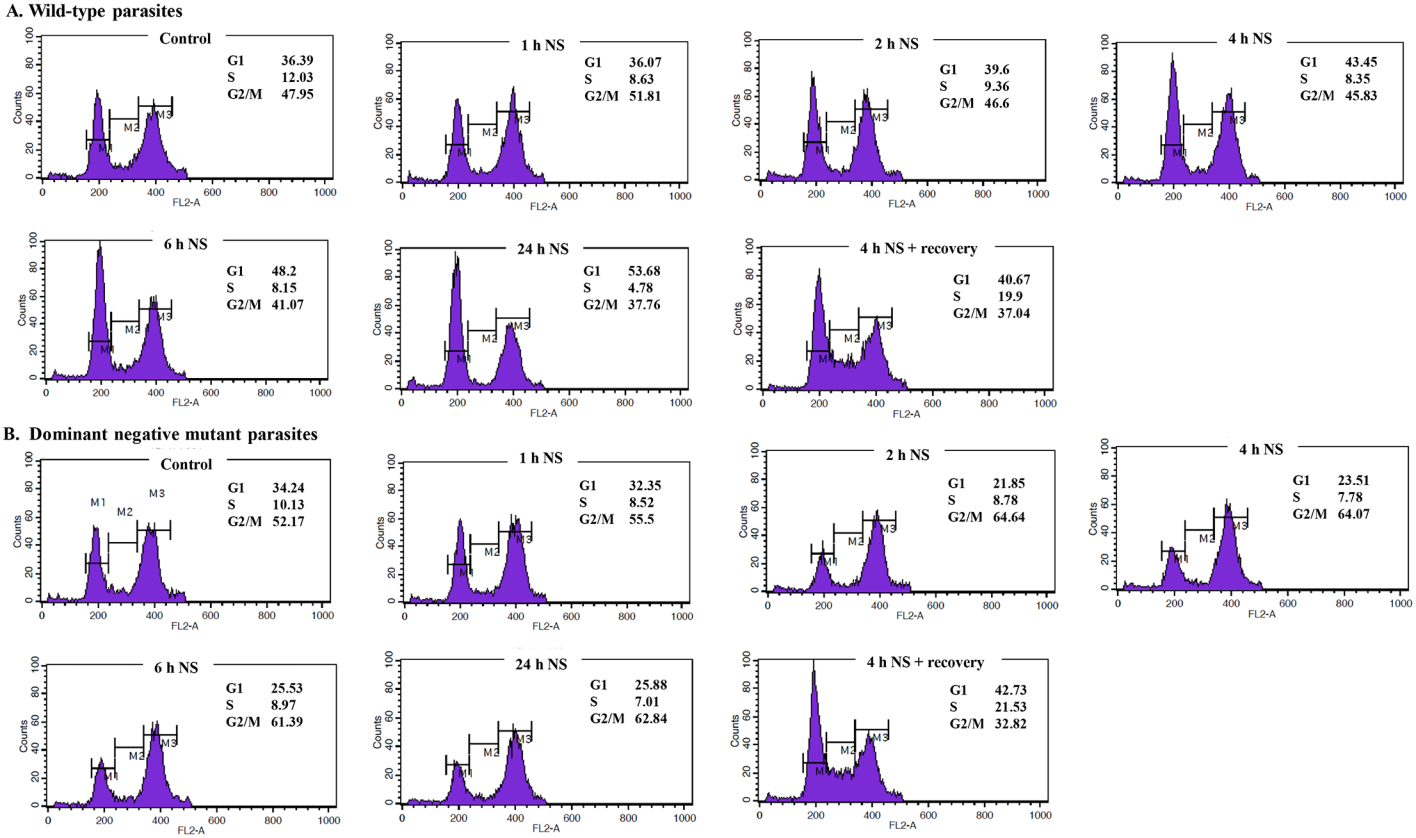
Cell cycle arrest in G1 phase during nutrient starvation is dependent on LdeK1 in *L*. *donovani*. *L*. *donovani* promastigotes were subjected to nutrient starvation for 1 h, 2 h, 4 h, 6 h and 24 h followed by fixation in 70% methanol and PI staining. The stained parasites were analyzed for cell cycle distribution by FACS using 10000 events. Nutrient deprivation induced a G1-arrest in wild-type parasites. However, parasites lacking a functional LdeK1 did not show an arrest in this phase of cell-cycle, further suggesting its role in nutrient starvation condition in *L*. *donovani*.

*L*. *donovani* promastigotes, both wild-type and the dominant-negative mutant were subjected to nutrient starvation for 1 h, 2 h, 4 h, 6 h and 24 h in EBSS. These parasites were fixed in ice-cold 70% methanol followed by PI staining and determination of cell-cycle distribution by FACS analysis. A G1 arrest was induced in the wild-type parasites after 2 h of nutrient starvation with maximum number of parasites in this phase by 24 h of stress (Fig 7A). Interestingly, in contrast, the dominant-negative mutant did not show any arrest in G1 phase, rather an increase in M-phase population was observed (Fig 7B). This indicates the role of LdeK1 in nutrient starvation in bringing about a G1-arrest possibly mediated by eIF2α phosphorylation. The expression of N-terminal domain of LdeK1 was analyzed by western blotting using anti-LdeK1 antibody. The expression is slightly reduced after 24 h of nutrient starvation in the dominant negative mutants. The reduction could be due to a general reduction in translation after 24 h of starvation (S4 Fig).

### Protein synthesis is affected in wild type parasites as compared to the dominant negative mutant during nutrient starvation

**Fig 8:**
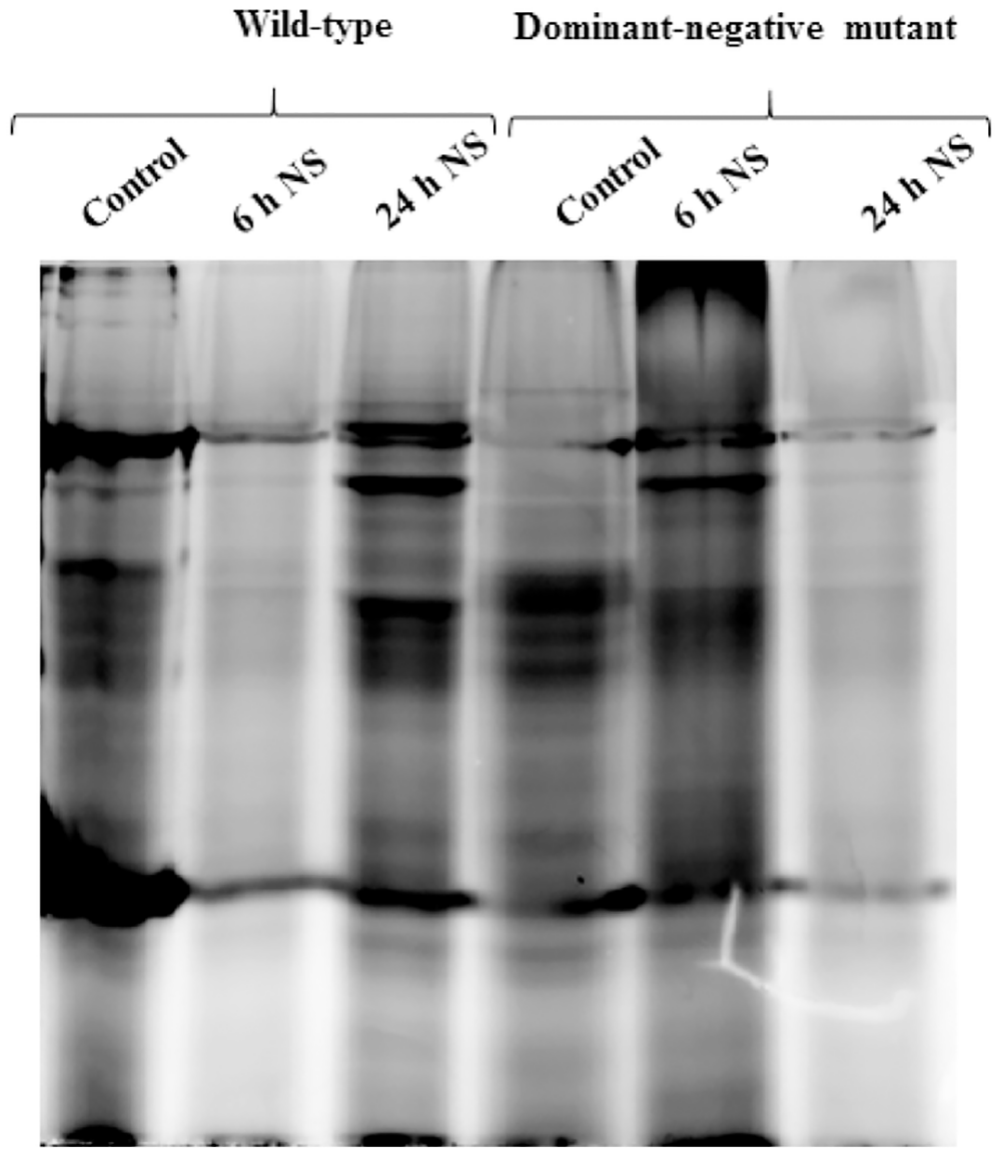
*De novo* protein synthesis is affected in wild type parasites as compared to the dominant-negative mutant (DNM) during nutrient starvation. Log-phase promastigotes both wild type and DNM were subjected to nutrient starvation for 6 h and 24 h followed by metabolic labelling of newly synthesized proteins using methionine analog L-azidohomoalanine (AHA). The protein synthesis is down regulated in the wild-type parasites at 6 h, however it is not affected in the DNM, indicating the role of LdeK1 in general protein synthesis.

*L*. *donovani* promastigotes, both wild-type and the dominant-negative mutant were subjected to nutrient starvation for 6 h and 24 h in EBSS. These including the control parasites were used for incorporation of AHA in the newly synthesized proteins. The nascent protein synthesized were labelled with fluorescent dye (TAMRA) followed by SDS-PAGE and detection in a laser scanner. The protein synthesis was down-regulated in the wild-type parasites after 6 h of nutrient starvation however, this down-regulation was not observed in case of DNM parasites (Fig 8) in correlation with a compromised eIF2α phosphorylation as described in Fig 6. This therefore establishes conclusively the role of LdeK1 in regulating protein synthesis during nutrient starvation.

## Discussion

Here, we report on cloning and characterization of an eIF2α kinase LDBPK_11.00060 from *L*. *donovani* which we named as LdeK1. It appears to be homologous to GCN2 of yeast and mammals. We have cloned the full length ORF of LdeK1 (3687 bp) that includes a RWD domain which is present upstream of the annotated start site in the *L*. *donovani* database. A GCN2-like kinase with an RWD-domain has been predicted in *T*. *brucei* (TbeIF2K1) and characterized in *T*. *gondii* (TgIF2K-D) [19,20]. However, PfeIK1 of *P*. *falciparum* and TgIF2K-C of *T*. *gondii* lack RWD domain though reported to respond to amino acid starvation [12,38]. The presence of GCN1 ortholog and the dominant-negative phenotype developed by overexpression of LdeK1NTD in *L*. *donovani*, suggest that GCN2 activation might depend on LdeK1-GCN1 interaction during translation regulation in these parasites like that in yeast [34]. The site of eIF2α phosphorylation by LdeK1 in *L*. *donovani* is T-166 as opposed to S-51 residue in mammals due to the presence of an additional N-terminal extension. A similar observation has also been reported for *T*. *brucei* [20], *T*. *cruzi* and *L*. *infantum* [22]. However, we report for the first time that a *Leishmania-*specific kinase LdeK1 is capable of phosphorylating the human substrate at S-51 residue *in vitro*. It is tempting to speculate that parasitic eIF2α kinases may be capable of phosphorylating their host eIF2α and down regulate host’s protein synthesis, a strategy common to parasite survival.

LdeK1 was found to be localized in the cytoplasm of *L*. *donovani* promastigotes as expected. This is in accordance with reports in other organism including *Toxoplasma* [16] and yeast [44]. The expression of LdeK1 at the transcript as well as the protein level was found to be down-regulated in axenic amastigotes of *L*. *donovani*, which is not surprising, because, the amastigotes are propagated in an environment which is considered to be nutrient-rich, where the requirement of GCN2 kinase is minimal.

GCN2 is known to get activated during nutrient starvation, a condition encountered by the parasites during their life inside the insect vector. PfeIK1 of *P*. *falciparum* and recently TgIF2K-C, of *T*. *gondii*, both GCN2-like kinases, were found to respond to nutritional stress. Phosphorylation of eIF2α of *L*. *donovani* in response to nutrient deprivation is a good indication of the possible involvement of a GCN2-like kinase in meditating nutrient stress response in this parasite (Fig 6). Our data, further confirms this by showing that eIF2α phosphorylation ability of the parasite in response to nutrient starvation is significantly impaired in parasites with compromised function of LdeK1 i.e., in the LdeK1 dominant negative mutant (Fig 6). Further, translation in the wild type is down-regulated at 6 h of nutrient starvation while it remains unaffected in the dominant negative mutants indicating a definitive role of LdeK1 in phosphorylation of eIF2α and subsequent protein synthesis arrest in response to this stress (Fig 8).

Nutrient starvation is known to induce cell-cycle arrest in G1 phase in budding yeast [45]. Furthermore eIF2α phosphorylation by GCN2 and PERK is known to cause a G1 arrest in response to unfolded protein response due to inhibition of cyclin D1 synthesis [46]. We demonstrate that G1-arrest occurs in response to nutrient starvation in *L*. *donovani*, which is dependent on LdeK1 activation, as *L*. *donovani* promastigotes expressing a dominant-negative mutant of LdeK1 failed to show a G1-arrest at this condition. The viability of the parasites however does not change during this stress condition. The cell morphology of Leishmanial parasites after starvation changes to narrow cell body with elongated flagellum which is a general phenomenon observed for *Leishmania* parasites during starvation conditions encountered by the parasite during stationary phase. This morphological change has been observed both in case of wild type and mutant. This result therefore indicates lack of any morphological difference between wild-type and the dominant-negative mutant (S5 Fig).

Attenuation of global translation leads to conservation of nutrients and preferential translation of specific transcripts required to cope up with the stress. eIF2α phosphorylation during amino acid starvation leads to preferential translation of transcription factors like GCN4 in yeast and ATF4 in mammals facilitating transcription of genes required for amino acid biosynthesis [10,12]. Survival from nutrient stress is highly significant in the life cycle of *Leishmania* like parasites, as these organisms have to cope up with nutrient deprivation that occurs within the midgut of sandfly vector during the time elapsed between two blood meals. Studies suggest that this nutrient stress actually induce metacyclogenesis, a process which transform the parasites into highly infective form [4,47]. During *in vitro* growth of *Leishmania*, nutrient starvation in stationary cultures caused promastigotes to exhibit metacyclogenic features [47]. Our data provides substantial evidence for the involvement of a GCN2-like kinase, LdeK1 in the translation regulation during nutrient stress and probably in the subsequent events of metacyclogenesis. In addition, the identification of many components involved in the GCN2 activation mechanisms downstream of nutrient deprivation and similar conditions, strongly suggest conservation of similar mechanism in this early diverged eukaryote. It is possible that the hyperphosphorylation of eIF2α may cause a switch from the global translation towards the translation of specific proteins which are involved in stress adaptive mechanisms and differentiation process as has been well-established in other organisms [11,16].

eIF2α phosphorylation was found to be more robust in hypervirulent strain than the hypovirulent strain of *T*. *gondii* which was also reflected in the reduced infection of eIF2α-S71A mutants [21]. It would therefore be interesting to determine whether LdeK1 and phosphorylation of eIF2α have any effect on the virulence of the *Leishmania* parasites. Taken together, our data along with the growing body of evidences recently accumulated in this area strongly suggest the importance of eIF2α kinases in the stress survival and differentiation process of protozoan parasites. Seemingly, these parasites are equipped with highly specific eIF2α kinases, which sense different environmental signals and collectively initiate adaptive response pathway which will help them to fight the adversity and ultimately differentiate into a new life form which is more adapted to live in the current environment. A G1 arrest in the cell cycle also accompanies gross morphological and biochemical remodelling, facilitating the crucial transformation process including differentiation into amastigotes [48]. Our data further suggests the dependence of LdeK1, the subsequent eIF2α phosphorylation and translation inhibition to provoke a G1-arrest during nutrient starvation. In *T*. *cruzi*, differentiation from epimastigotes to infective metacyclic trypomastigotes induced by nutritional starvation required phosphorylation of eIF2α and down regulation of protein synthesis [24]. Our study suggests that nutrient starvation experienced by the promastigotes in the vector might activate LdeK1 in *L*. *donovani* causing eIF2α phosphorylation and the subsequent G1-arrest in the cell cycle. We therefore propose that translation regulation mediated by eIF2α phosphorylation by LdeK1 and a G1-arrest in cell cycle could probably be parts of an attempt by the *Leishmania* promastigotes to retool their cellular dynamics that may pave the way towards their metacyclogenesis.

## Supporting Information

S1 Fig Cloning of full length LdeK1 from *L*. *donovani*. **(A)** PCR amplification of LdeK1 from *L*. *donovani*. Genomic DNA of *L*. *donovani* was used as the template to amplify the LdeK1. Lane 1, 1kb gene ruler; lane 2, phusion polymerse amplified kinase domain of LdeK1. **(B)** Confirmation of pGEMT-LdeK1 by Colony PCR. Among 7 colonies screened (1–7), 4 were positive. Negative control (without template), positive control (genomic DNA used as template). **(C)** Plasmids were isolated from screened clones and analyzed by restriction digestion using *EcoR*I. Lane 1, 1kb gene ruler; lanes 2 and 4, uncut pGEMT-LdeK1; lanes 3 and 5, *EcoR*I digested plasmids showing the released insert (3687 bp).
(TIF)

S2 Fig Cloning, overexpression and purification of kinase domain of LdeK1. **(A)** PCR amplification of catalytic domain of LdeK1 from *L*. *donovani*. Genomic DNA of *L*. *donovani* was used as the template to amplify the catalytic domain of LdeK1. Lane 1, 1kb gene ruler; lane 2–5, phusion polymerse amplified kinase domain of LdeK1. **(B)** Confirmation of pET28a-LdeK1KD by restriction digestion analysis. Plasmids were isolated from screened clones and analyzed by restriction digestion using *EcoR*I and *Not*I. Lane 1, 1kb gene ruler; lane 2, uncut pET28a-LdeK1KD; lane 3, *EcoR*I & *Not*I digested plasmid showing the released insert (1164 bp). **(C)** 10% SDS PAGE profile of the overexpressed catalytic domain of LdeK1 in *E*. *coli*. Lane 1, molecular weight marker; lane 2, uninduced cell fraction; lane 3, induced fraction of *E*. *coli*; lane 4, isolated inclusion bodies. (**D**) 10% SDS PAGE profile of Ni-NTA purification of LdeK1KD. Lane 1, molecular weight marker; lane 2, flow through; lane 3, wash 1; lane 4, wash 2, lane 5, elute 1; lane 6, elute 2; lane 7, elute 3.
(TIF)

S3 Fig *In vitro* phosphorylation of LdeIF2α is mainly due to catalytic domain of LdeK1. To exclude the possibility of any contaminating kinase in the preparation of the bacterially overexpressed catalytic domain of LdeK1 (LdeK1KD), the lysates of untransformed and transformed Rosetta cells were incubated with wild-type LdeIF2α along with cold ATP at 30°C and subjected to SDS-PAGE. The phosphorylation of eIF2α was analyzed by western blotting using anti-phospho-eIF2α antibody. Leishmanial total eIF2α was used as loading control.
(TIF)

S4 Fig The expression of N-terminal domain of LdeK1 is slightly reduced after 24 h of nutrient starvation. The expression of N-terminal domain of LdeK1 in nutrient starved DNM parasites were analyzed by western blotting using anti-LdeK1 antibody. The expression is slightly reduced at 24 h of nutrient starvation.
(TIF)

S5 Fig Morphology of wild-type and DNM parasites changes during nutrient starvation. The morphology of both wild type and DNM changes to parasites with a narrow cell body and a longer flagellum after 24 h of nutrient starvation indicating lack of any morphological difference between wild-type and the dominant-negative mutant.
(TIF)
