# Supplementary figures and images for: A GCN2-Like eIF2α Kinase (LdeK1) of Leishmania donovani and Its Possible Role in Stress Response
Source: PLoS One. 2016 Jun 1;11(6):e0156032. doi: 10.1371/journal.pone.0156032 (PMC4889150; doi:10.1371/journal.pone.0156032)

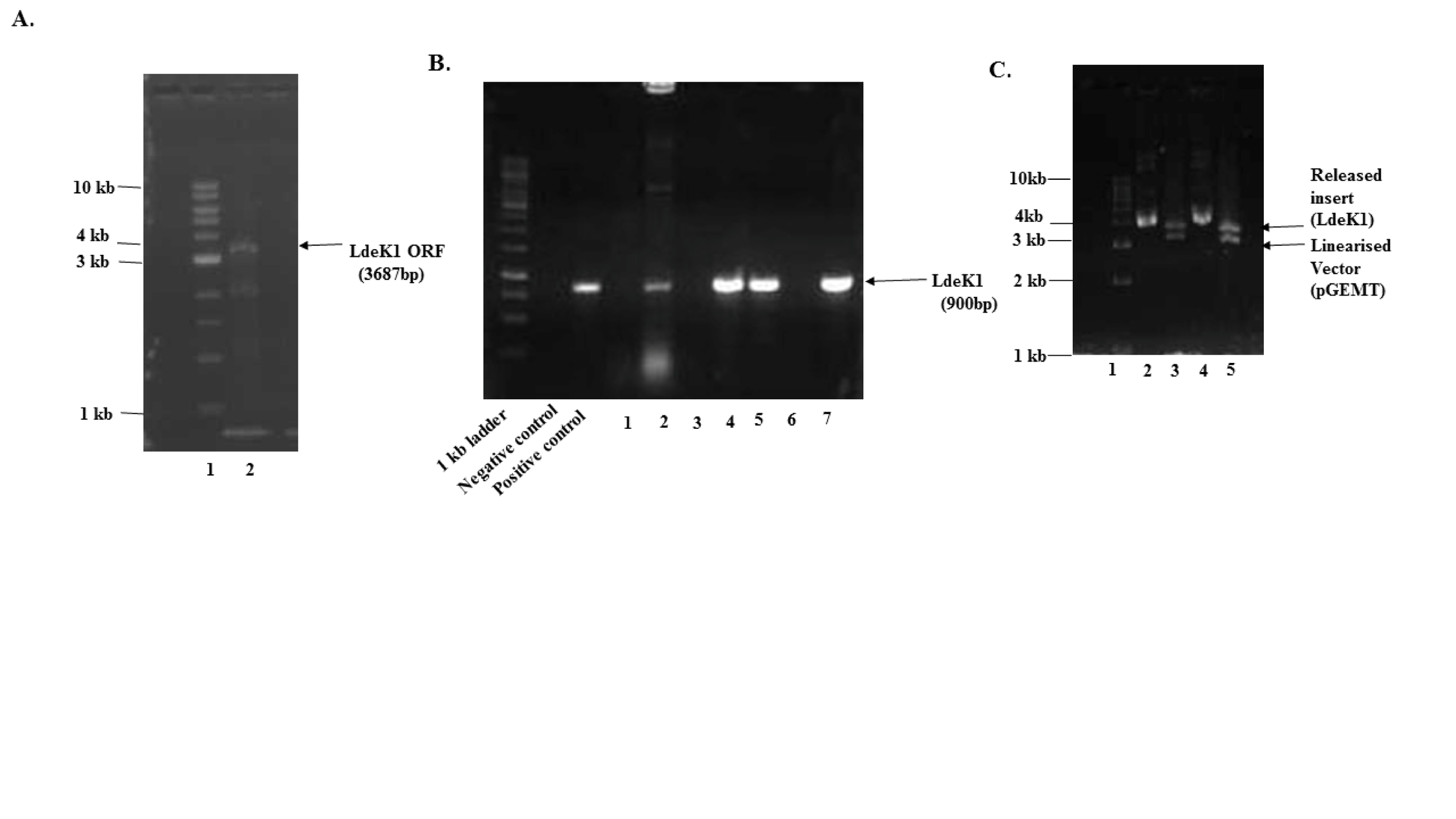

Supplement: S1 Fig — (A) PCR amplification of LdeK1 from L. donovani. Genomic DNA of L. donovani was used as the template to amplify the LdeK1. Lane 1, 1kb gene ruler; lane 2, phusion polymerse amplified kinase domain of LdeK1. (B) Confirmation of pGEMT-LdeK1 by Colony PCR. Among 7 colonies screened (1–7), 4 were positive. Negative control (without template), positive control (genomic DNA used as template). (C) Plasmids were isolated from screened clones and analyzed by restriction digestion using EcoRI. Lane 1, 1kb gene ruler; lanes 2 and 4, uncut pGEMT-LdeK1; lanes 3 and 5, EcoRI digested plasmids showing the released insert (3687 bp). (TIF) [file pone.0156032.s001.tif]

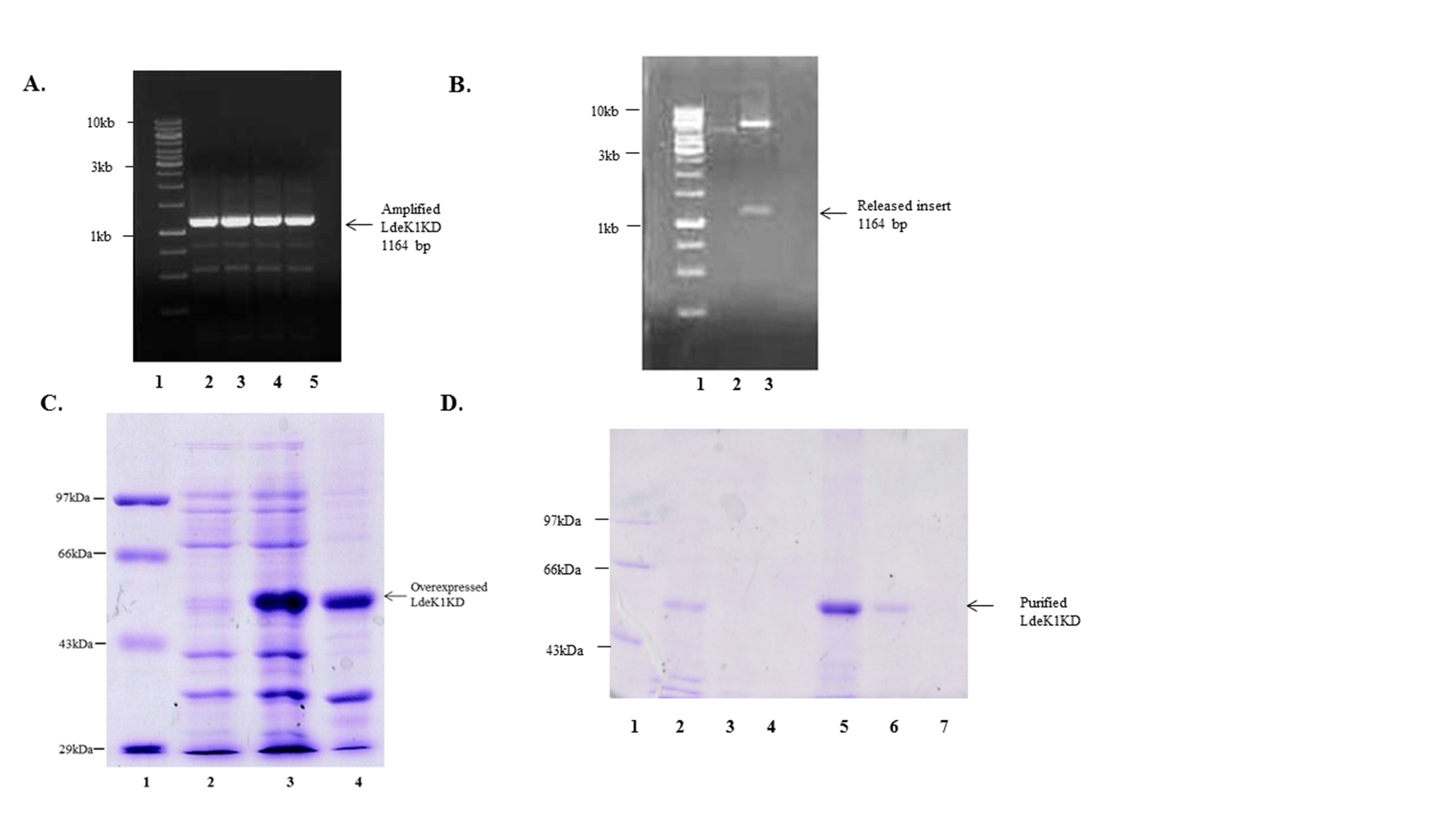

Supplement: S2 Fig — (A) PCR amplification of catalytic domain of LdeK1 from L. donovani. Genomic DNA of L. donovani was used as the template to amplify the catalytic domain of LdeK1. Lane 1, 1kb gene ruler; lane 2–5, phusion polymerse amplified kinase domain of LdeK1. (B) Confirmation of pET28a-LdeK1KD by restriction digestion analysis. Plasmids were isolated from screened clones and analyzed by restriction digestion using EcoRI and NotI. Lane 1, 1kb gene ruler; lane 2, uncut pET28a-LdeK1KD; lane 3, EcoRI & NotI digested plasmid showing the released insert (1164 bp). (C) 10% SDS PAGE profile of the overexpressed catalytic domain of LdeK1 in E. coli. Lane 1, molecular weight marker; lane 2, uninduced cell fraction; lane 3, induced fraction of E. coli; lane 4, isolated inclusion bodies. (D) 10% SDS PAGE profile of Ni-NTA purification of LdeK1KD. Lane 1, molecular weight marker; lane 2, flow through; lane 3, wash 1; lane 4, wash 2, lane 5, elute 1; lane 6, elute 2; lane 7, elute 3. (TIF) [file pone.0156032.s002.tif]

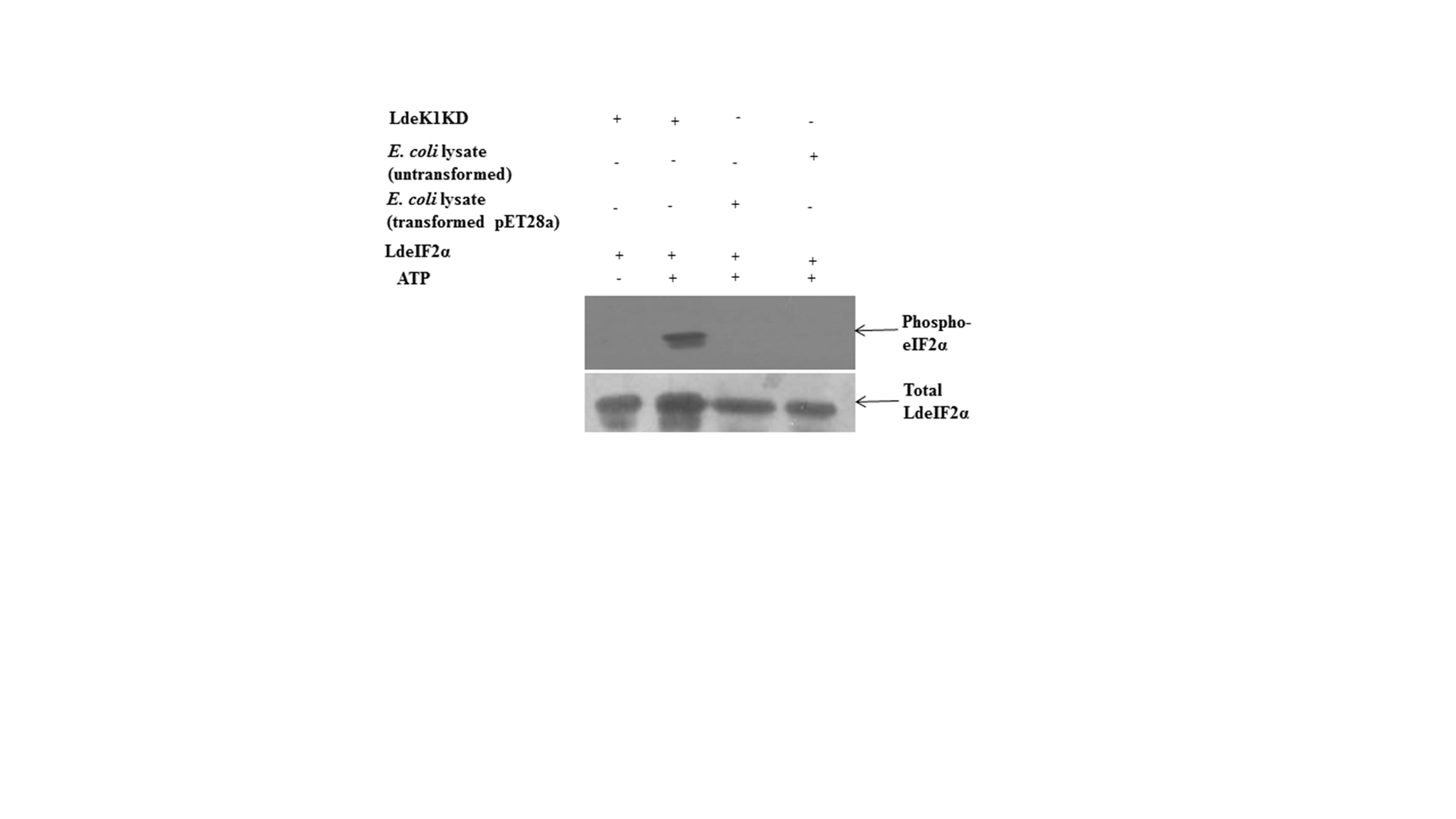

Supplement: S3 Fig — To exclude the possibility of any contaminating kinase in the preparation of the bacterially overexpressed catalytic domain of LdeK1 (LdeK1KD), the lysates of untransformed and transformed Rosetta cells were incubated with wild-type LdeIF2α along with cold ATP at 30°C and subjected to SDS-PAGE. The phosphorylation of eIF2α was analyzed by western blotting using anti-phospho-eIF2α antibody. Leishmanial total eIF2α was used as loading control. (TIF) [file pone.0156032.s003.tif]

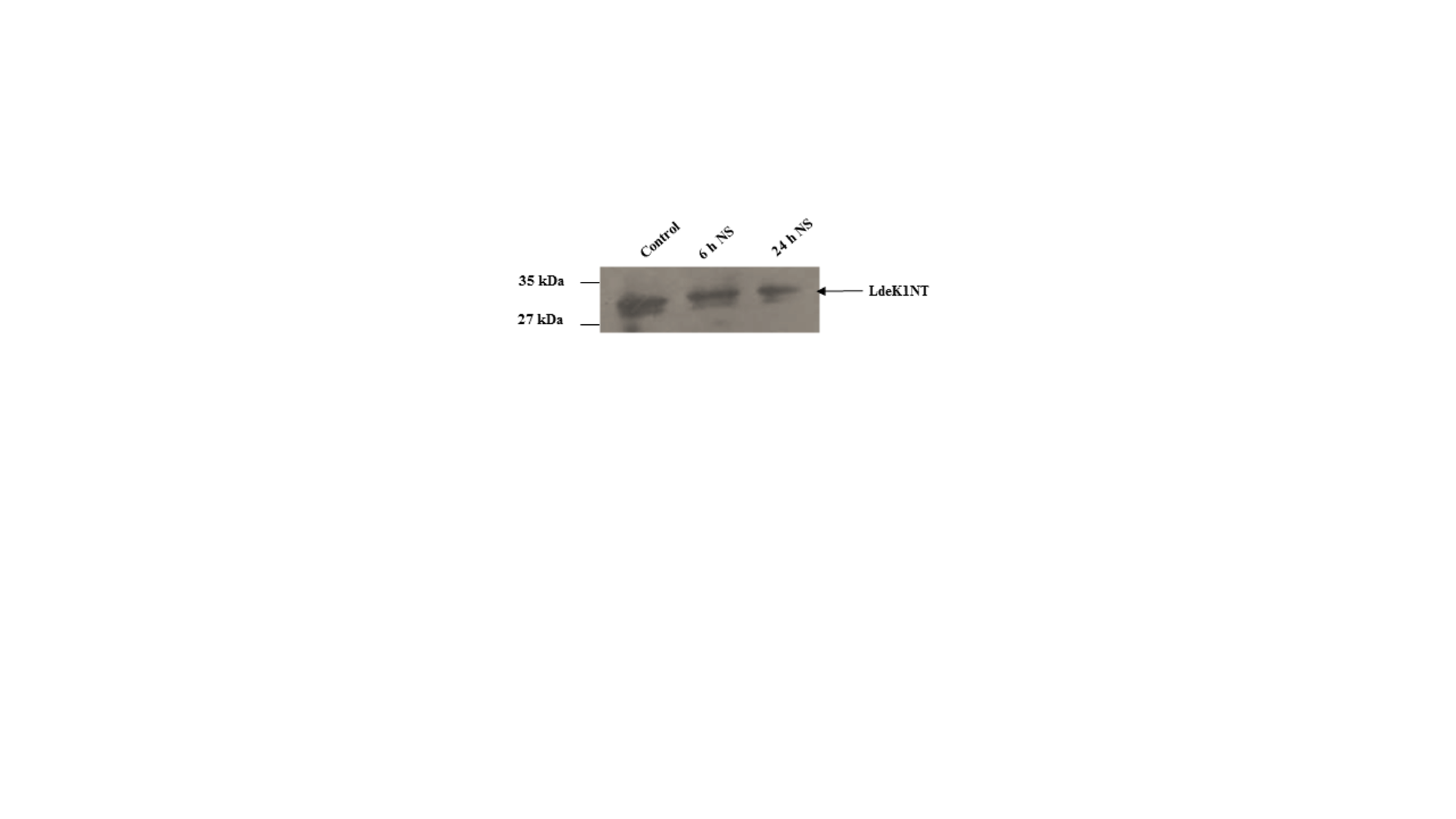

Supplement: S4 Fig — The expression of N-terminal domain of LdeK1 in nutrient starved DNM parasites were analyzed by western blotting using anti-LdeK1 antibody. The expression is slightly reduced at 24 h of nutrient starvation. (TIF) [file pone.0156032.s004.tif]

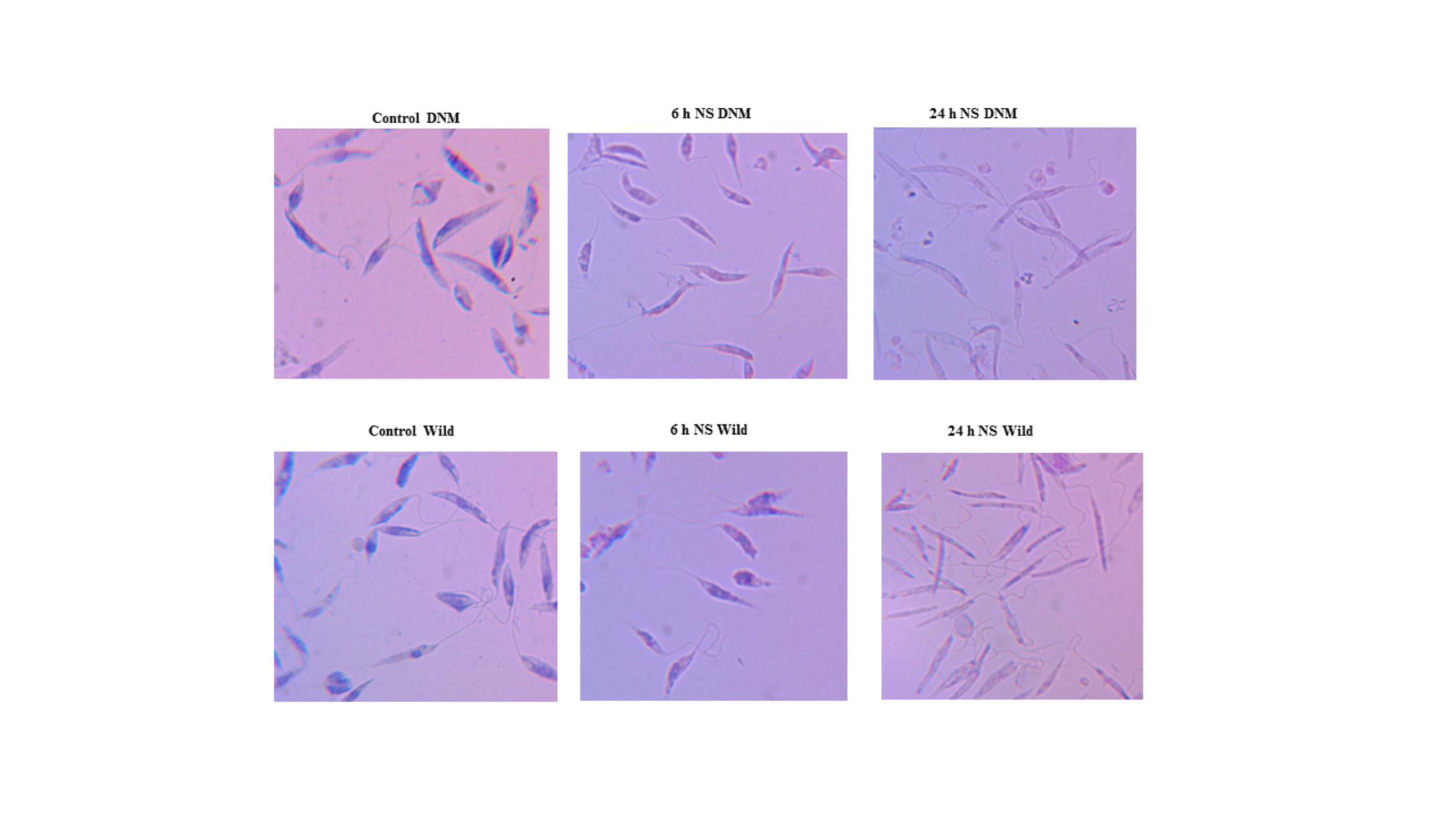

Supplement: S5 Fig — The morphology of both wild type and DNM changes to parasites with a narrow cell body and a longer flagellum after 24 h of nutrient starvation indicating lack of any morphological difference between wild-type and the dominant-negative mutant. (TIF) [file pone.0156032.s005.tif]
